# Hydrothermal Liquefaction of Biomass as One of the Most Promising Alternatives for the Synthesis of Advanced Liquid Biofuels: A Review

**DOI:** 10.3390/ma14185286

**Published:** 2021-09-14

**Authors:** Lucía Grande, Ivan Pedroarena, Sophia A. Korili, Antonio Gil

**Affiliations:** INAMAT^2, Departamento de Ciencias, Campus de Arrosadía, Universidad Pública de Navarra, 31006 Pamplona, Spain; lucia.grande@unavarra.es (L.G.); ivan.pedroarena@unavarra.es (I.P.); sofia.korili@unavarra.es (S.A.K.)

**Keywords:** biomass, biofuels, biowastes, bioprocesses, chemical methods, biochemical methods, thermochemical methods

## Abstract

The use of biofuels offers advantages over existing fuels because they come from renewable sources, they are biodegradable, their storage and transport are safer, and their emissions into the atmosphere are lower. Biomass is one of the most promising sustainable energy sources with a wide variety of organic materials as raw material. Chemical, biochemical, and thermochemical methods have been proposed to obtain biofuels from raw materials from biomass. In recent years, a thermochemical method that has generated great interest is hydrothermal liquefaction. In this paper, a brief review of the main sources for liquid biofuels and the synthesis processes is presented, with special emphasis on the production of biofuels using hydrothermal liquefaction by using waste generated by human activity as raw material.

## 1. Introduction

In the early 20th century, biomass from crops and trees was used as a starting material to produce dyes, solvents, or synthetic fibers. In addition, biochemical and chemical research were promoted to study the production of biofuels from biomass. However, by the end of the 1960s, most of these products had been displaced by petroleum derivatives. The energy crisis of 1970 reactivated research concerning this type of process, but the subsequent fall in the price of fuel once again reduced the interest of society [1]. Nowadays, the environmental damage caused by fossil fuels and the increasingly imminent climate change have driven the development of renewable energies as well as the search for new energy sources. In this context, biofuels offer several advantages compared to existing fuels, since they are biodegradable, their storage and transport is safer, and their emissions are lower [2].

Biofuels can be produced from several raw materials. For example, a type of biofuel to have generated interest recently would be those formed from Polyhydroxyalkanoates (PHA), which are structurally diverse microbial polyesters synthesized by numerous bacteria. From this novel substrate, a biofuel is obtained similar to biodiesel, except for it having a high oxygen content and no nitrogen or sulfur [3,4]. Biomass is also presented as one of the most promising sustainable energy sources. A wide variety of organic materials, such as forest and agricultural residues, aquatic plants, energy crops, sewage sludge, or leftover food, could be used as energy sources and help to reduce greenhouse gas emissions and dependence on fossil fuels [1,5]. In addition, the generation of waste is increasing with the world population. Therefore, biomass will eventually become a more abundant and sustainable energy source than traditional fossil fuels, whose availability is gradually decreasing more and more [6]. On the other hand, despite the fact that biofuels generated from microalgae will not be analyzed in depth in this review, it is necessary to comment that there are more and more studies that propose this type of raw material for the generation of sustainable liquid fuels [7].

Biofuels generated from plant biomass have various advantages. First of all, the uptake of CO_2_ that occurs during photosynthesis of plants compensates for the CO_2_ that is released during the combustion of biofuel, so it does not increase the net CO_2_ in the atmosphere. Besides, the production of these types of bioproducts can generate new income and employment opportunities in rural areas [8]. One of the main disadvantages of biomass from plants is that availability and productivity decrease in winter [9]. To overcome this problem, hydrothermal liquefaction (HTL) plants are being developed that use other types of raw materials, such as the biowaste that humans generate or algae that are easily cultivable [9]. Hydrothermal liquefaction began to generate interest in the 1960s, when Appell et al. developed a process for the liquefaction of cellulosic biomass [10] and waste biomass [11]. Later, in the 1980s, the hydrothermal upgrading (HTU) process was developed and the construction of a pilot plant was promoted. In addition, the hydrodeoxygenation process was proposed as a method to upgrade the produced bio-crude to fuel [12]. In 1986, the STORS (sludge-to-oil reactor system) process [13] was proposed for direct hydrothermal liquefaction by the United States Environmental Protection Agency (USEPA). This process was conceived for the direct continuous thermochemical liquefaction of primary municipal sewage sludge. In this way, technology has been gaining importance to become one of the most hopeful strategies to generate sustainable fuels [2,6].

The development and optimization of new biofuels is a field that is strongly promoted, since it is an essential technology for the sustainable development of society. Therefore, the number of papers focused on the various types of biofuel, raw materials, and generation processes is high. Especially in the case of hydrothermal liquefaction, as it is a technique that provides very good results, scientific research in this field has increased exponentially in the last five years. Taking into account the amount of information available in the open literature, it is essential to filter all these contributions and collect the most important ones, in order to be able to assess the current state of the art.

## 2. Biofuels

Biofuels are fuels used in means of transport in liquid or gaseous states, such as biodiesel and bioethanol, which are obtained from biomass [8]. Various classifications for biofuels can be found in the literature, the most common way being to differentiate three generations based on their raw material sources [14,15]. First generation generally refers to biofuels produced from human food-based raw materials [8]. For example, biodiesel is produced from edible vegetable oils and animal fats through a transesterification reaction [16]. First generation biofuel production technologies are well established, however competition with the food supply chain is under debate. It has been suggested that the increased use of food crops for energy production could raise food prices [17]. In addition, another factor to take into account is the possibility that, in the future, the effects of climate change could decrease the yield of food crops [18]. To overcome this problem, the use of advanced second and third generation biofuels has been proposed, i.e., biofuels that use raw materials that do not compete with food and that may have better environmental indicators. Second generation biofuels are produced from non-food energy crops (straw, agricultural waste, etc.), in particular lignocellulosic materials, through more advanced manufacturing processes than those for the first generation. Despite the fact that it is a highly studied energy source, the complexity of the processes and the scarce availability of raw materials limit its potential. The third generation is made up of fuels obtained from microorganisms, such as microalgae and bacteria [14,15].

The main advanced liquid biofuels are the following:

Biodiesel: Biodiesel is the best alternative fuel in compression ignition engines. No modification is required when using a biodiesel blend (up to 20%) with diesel fuel in these types of engines. For pure biodiesel, it is necessary to carry out some modifications to the engines [15]. The second generation of this type of fuel is obtained from inedible oils and the third generation from waste oils and algae, which are abundant in nature and often available locally. At high temperatures and in the presence of oxygen, biodiesel tends to oxidize [19]. Because of these stability and decomposition problems, biodiesel is generally mixed with conventional diesel [15].

**Figure 1:**
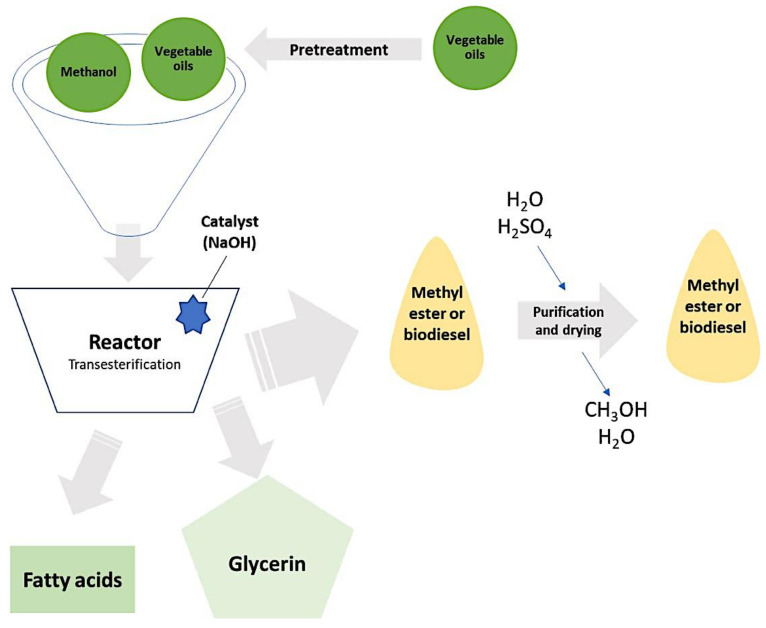
Biodiesel manufacturing process. Adapted from references [18,19].

One of the problems posed by second-generation biodiesel is that despite the fact that the raw material needed to generate this type of fuel does not have food functions, crops compete for land with crops intended to feed the population. Furthermore, the biodiesel production of this generation requires an excessive volume of alcohol [19]. To address these types of socio-economic difficulties, researchers are working on a modern, cost-effective, and readily available alternative approach. In the bibliography, detailed descriptions of the biodiesel manufacturing process can be found [19,20]. A general summary diagram of this manufacturing process is included in Figure 1.

The objective of this first step is to eliminate components such as water, suspended particles or free fatty acids. For seeds with a high level of oil content, the most widely used method is one that combines mechanical treatment (pressing using screw presses) and the use of solvents [21,22]. The pretreatment process is usually carried out continuously and the systems can be classified as immersion, percolation, or mixed immersion–percolation. In immersion systems, the raw materials are directly immersed in the solvent in a countercurrent flow, whereas in percolation systems, the solvent is moved rapidly over the surface of the raw material. This system achieves a mixture rich in oil (up to 35 wt.%) [23]. The refined oils and an alcohol such as methanol are then introduced into the reactor and the transesterification reaction occurs. This reaction can be catalyzed by an acid or by a base, with the most commonly used catalyst being NaOH. As a product of the reaction, biodiesel is obtained, which has to be purified for later use as a biofuel. In addition, the transesterification reaction gives rise to other products, such as fatty acids or glycerin, which are used in the food industry and in the cosmetic industry [20,24,25].

Hydrotreated vegetable oil (HVO) or green diesel: This is a new generation biodiesel that does not contain oxygen, aromatic compounds, and sulfur. It is possible to generate it thanks to two types of technology, namely hydroprocessing and thermochemical processes, such as pyrolysis and gasification. Green diesel has a higher cetane number than biodiesel, this increases thermal efficiency and reduces fuel consumption. Furthermore, the calorific value of green diesel is higher than the calorific value of conventional diesel and biodiesel. Lastly, HVO has a higher oxidative stability than fatty acid methyl ester. However, its lubrication capacity is lower, and its CO_2_ emissions are higher [15,26,27,28,29]. Table 1 presents a comparison of the characteristics of conventional diesel, biodiesel, and green diesel.

Bioethanol: This is the most widely used biofuel. It is obtained through the hydrolysis of cellulose present in lignocellulosic biomass (such as wood, straw, or grass) and the fermentation of sugars [30]. This process occurs in two steps. Firstly, the fermentation of carbohydrates by yeast occurs, which gives rise to hydrated ethanol. In the second step, residual water is removed by adsorption, extractive distillation, or membrane separation to obtain anhydrous ethanol [31]. In Europe, the most used blend is E10, which is made up of 10% ethanol and 90% conventional gasoline. However, superethanol E85 (85% ethanol) is on the rise and cars that use this fuel are becoming more and more common [32].

Synthetic liquid biofuels: Several thermochemical routes, such as pyrolysis, gasification, and liquefaction, can convert biomass into synthetic liquid fuels [33]. Synthetic biofuels have similar or even superior properties to their fossil counterparts. They contain virtually no sulfur or aromatics and can be used in existing engines without any technological modification [34].

Biobutanol and biopentanol: These alcohols have some advantages over other shorter chain alcohols such as ethanol. Among these advantages, the higher energy density and less hygroscopic nature can be highlighted. Moreover, the cetane number is better, the flash point is higher, and the autoignition temperature is lower. It is possible to mix them with conventional diesel and use the mixture in a compression ignition engine. These blends have lower viscosities, higher oxygen content, and better ignition quality, characteristics that help achieve better atomization and combustion [35]. Butanol and pentanol can be obtained by the fermentation of biomass or the biosynthesis of glucose by the action of microorganisms and cyanobacteria. The biggest drawbacks for the fermentation of these alcohols are low concentration and limited productivity, along with the price of raw materials [36].

## 3. Raw Materials for the Synthesis of Biofuels

Annually, approximately 100 billion metric tons of biomass waste are produced in the world [37]. In the past, these residues were burned or turned naturally into organic fertilizers under favorable conditions. However, biowaste can cause serious health and environmental problems if not treated properly. The great majority of environmental problems come from the generation of greenhouse gases (GHG), mainly methane emissions [38,39]. The reduction of stratospheric ozone also occurs due to the generation of emissions of halogenated compounds contained in biowaste such as volatile organic compounds (VOC), nitrogen oxides and sulfur oxides [38]. Health problems are produced by the exposure of chemical products during the waste collection and treatment stages, as well as by the addition of heavy metals to surface waters and soil [40]. In fact, in underdeveloped countries, the burning of agricultural waste is a very common practice that releases gases, such as carbon monoxide, nitrogen dioxide, and nitrous oxide, among others [41].

Biomass can be classified into three large groups: natural biomass, which is produced without human intervention; residual biomass that comes from human activity; and biomass produced with the sole objective of its energy use. This study is focused on residual biomass, and more specifically on three specific types of biowaste: the organic fraction of urban solid waste (OFUSW); sludge from wastewater treatment plants (WWTP) and biomethanization digestate. OFUSW has a specific heat capacity of 2.72 kJ/(kg·K) and the digestate has a specific heat capacity of 4.14 kJ/(kg·K) [42]. The specific heat of the sludge depends on the water content present. It varies between 1.23 kJ/(kg·K) and 4.18 kJ/(kg·K) (specific heat of water) [43]. Therefore, without considering other parameters, the sludge from the treatment plants, and especially the digestate, seem to represent a more interesting energy source than OFUSW.

### 3.1. Organic Fraction of Urban Solid Waste (OFUSW)

Organic fraction of urban solid waste (OFUSW) is understood to be the part of urban solid waste constituted by those of organic origin, such as food remains, manure, tree pruning, street sweeping, branches, straw, and plants [44]. The organic fraction of organic solid waste represents 50% of this waste and contains mainly carbohydrates (30–40%), lipids (10–15%), and proteins (5–15%) [45].

Urban solid waste (USW) is a very heterogeneous material whose generation rate and composition depend on the place and the annual season. In order to accurately estimate the composition and average quantities of urban waste, it is necessary to perform a statistical sampling [46]. Representatively, an example of composition is included in Table 2. The average amount of USW that a person generates in Europe is 475 kg. Each year, 1.3 billion tons of urban solid waste are produced worldwide [45,47], and it is estimated that, in 2025, production will amount to 2.2 billion tons per year with an organic content of approximately 46% [48]. The characteristics and composition of OFUSW decisively affect the quality of the biofuel produced from them. However, the heterogeneity of these substances makes their characterization difficult [49].

### 3.2. Sludge from Wastewater Treatment Plants (WWTP)

The sludge from WWTP is solid, semi-solid or liquid waste with a muddy appearance that is produced after the treatment of domestic and industrial wastewater. Sludge contains a variety of organic and inorganic compounds. Organics include detergents, pesticides, fats, oils, dyes, solvents, or phenols [50]. The sludge composition is highly variable and depends on the pollution load of the waste water from which it comes, as well as the technical characteristics of the waste plant where its treatment is carried out. The sludge contains materials with agronomic value (organic matter, phosphorus, nitrogen, potassium, magnesium, calcium, and other micronutrients for plants), and other potentially polluting materials such as heavy metals such as lead, mercury, cadmium, covers, chromium, nickel and zinc, pathogens, and organic pollutants [51]. A WWTP that has an activated sludge process produces two main types of sludge: primary sludge and secondary sludge. The primary sludge is a combination of floating grease and solids collected at the bottom of the primary settler. Regarding the secondary or activated sludge, it is made up of microbial cells and suspended solids collected in the secondary settler.

### 3.3. Biomethanization Digestate

Biomethanization or anaerobic digestion is a biological process that takes place in the absence of oxygen and in several stages with the intervention of heterogeneous populations of microorganisms. The objective is to transform the most degradable fraction of organic matter into biogas, a mixture of gases made up mainly of methane, carbon dioxide, and other gases to a lesser extent (CO, N_2_, H_2_, water vapor, H_2_S) [46]. The matter resulting from this process, which is called biomethanization digestate, can be considered a waste or can be treated again to obtain another type of biofuel [52,53,54].

## 4. Advanced Biofuel Production Processes

The conversion of solid biomass into liquid fuels is not a spontaneous process [55,56]. Biomass raw materials can be processed by chemical, biochemical, and thermochemical methods.

### 4.1. Chemical and Biochemical Methods

Biochemical conversion processes use biological agents, such as bacteria or enzymes (other catalysts in the case of chemical methods), to break down biomass and give rise to available carbohydrates, which can be converted into liquid fuels and biogas, as well as various types of bioproducts [57]. The most widely used biochemical methods in the generation of advanced biofuels are fermentation and anaerobic digestion [58]. Instead, the most commonly used chemical processes are esterification and transesterification [58].

#### 4.1.1. Anaerobic Digestion

Anaerobic digestion is a process involving a series of chemical reactions that occur through natural metabolic pathways, enabled by microorganisms in an oxygen-free environment. The process is divided into four stages: hydrolysis, acidogenesis, acetogenesis, and methanogenesis. These reactions decompose organic macromolecules, giving rise to simpler molecules that lead to the generation of biogas and digestate [59,60].

#### 4.1.2. Fermentation

The fermentation process of organic materials consists of a series of biochemical reactions, the objective of which is the conversion of simple sugars, such as hexoses and pentoses, into ethanol and CO_2_, under anaerobic conditions by the action of microorganisms. The raw material used in fermentation processes is classified into three different classes: sugars, starch, and lignocellulosic substrates [59]. The performance of the process and the quality of the products generated depend on several factors, such as raw material, temperature, pH, and fermentation time [61].

#### 4.1.3. Transesterification

The chemical transformation of biomass leads to the production of high-density biofuels. More specifically, biodiesel is obtained from various types of vegetable oils (including lipids from algae) and animal fats through the transesterification process [59,62]. Transesterification or alcoholysis is a reversible reaction that produces esters and glycerol when fatty acids, alcohol, and an acid or base catalyst are mixed [19].

### 4.2. Thermochemical Methods

As the name itself indicates, in the thermochemical conversion of biomass energy is produced thanks to heat and chemical processes. Four types of thermochemical processes are distinguished: gasification, pyrolysis, combustion, and liquefaction. A comparison of the advantages and disadvantages of gasification, pyrolysis, and combustion processes are included in Table 3. Liquefaction is discussed more extensively later.

#### 4.2.1. Gasification

The raw material that can be used for gasification is very heterogeneous: from agricultural, lignocellulosic, forestry wastes or OFUSW, as long as the moisture content of the latter is less than 40%. The gasifying agents can be air, pure oxygen, steam, carbon dioxide, or mixtures. Air is the most economical and widely used agent, however it contains a considerable amount of nitrogen that decreases the heating rate of the synthesis gas obtained. Pure oxygen produces higher heating rates, but the cost of oxygen production lowers the economic profitability of the process. The rate of heating and the hydrogen contained in the synthesis gas can be increased if steam is used as the gasifying agent, in which case the rate of heat is increased to 10–15 MJ/Nm^3^, as compared to 3–6 MJ/Nm^3^ obtained from biomass gasification using air as agent. Processes using CO_2_ as agent are promising due to its presence in synthesis gas [64]. Therefore, it is evident that the chemical composition of the synthesis gas will vary substantially depending on the raw material used and the gasifying agent used.

Depending on the configuration, gasifiers are classified into three main types: fixed bed, fluidized bed, and entrained flow [65]. Fixed-bed gasifiers are ideal for small-scale biomass use. Fluidized bed gasifiers are used to treat biomass and Fuel Derived from Waste (FDW). These fluidized bed gasifiers are in turn divided into circulating or bubbling type. Circulating type beds are more used for biomass, while bubbling type beds are used to treat FDW [66]. Finally, entrained flow gasifiers require raw material with a micrometric order of magnitude and they are used for the processing of coal and in some cases the co-processing of coal and biomass.

#### 4.2.2. Pyrolysis

Pyrolysis is a thermochemical decomposition process that converts organic materials into solid biochar, bio-oil, and pyrolytic gas through the application of heat under anaerobic conditions. Pyrolysis consists of multiple spontaneous reactions whose efficiency is affected by factors such as temperature, heating rate, residence time, particle size, pressure, type of biomass, moisture content, and the pretreatment method of the biomass [67]. There are four types of pyrolysis.

Slow pyrolysis: This process uses very slow heating rates (<10 °C/min), with temperatures of 400 °C and residence times that can be up to several days. Traditionally used when charcoal is the target product. In general, slow pyrolysis provides high solid and low liquid yields [67].

Intermediate pyrolysis: Intermediate pyrolysis is used to obtain a combination of slow and fast pyrolysis products. In general, the temperature conditions are between 300–600 °C and speeds of 0.1 °C/min to 10 °C/min. The advantage of this process is that different particle sizes can be used [67].

Fast pyrolysis: Typically, fast pyrolysis gives high yields for liquids with low yields for solids. It involves the use of very fast heating rates, a very short residence time and rapid cooling to obtain bio-oil with high yields. Bio-oil is the target product of rapid pyrolysis with yields up to 70–80% [67].

Instant pyrolysis: Flash pyrolysis has won popularity as a process for the production of liquid fuels from biomass using short reaction times and very high temperatures in order to avoid the re-polymerization of decomposed products [68]. With this process, yields above 75% of bio-oils are achieved [69].

#### 4.2.3. Combustion

Nowadays, combustion processes produce a large part of the renewable energy obtained from biomass. Combustion plants can operate with several types of biomass, such as wood, dry leaves, hard vegetable husks, rice husks or dried animal manure [70]. The combustion process consists of an exothermic chemical reaction. Biomass is burned in the presence of air with the consequent release of chemical energy. Combustion takes place inside combustion chambers at temperatures between 800 and 1000 °C. It is important to highlight that the biomass used to obtain biofuels by combustion must have a humidity percentage lower than 50% [59].

#### 4.2.4. Hydrothermal Liquefaction

The thermochemical liquefaction of biomass is the process that has generated the most interest in recent years since it generates higher energy density, requires a shorter reaction period, and can be applied to a wider range of materials [55,71]. In addition, the liquid product resulting from techniques such as pyrolysis generally has a high oxygen content, which increases instability and makes it difficult to use as a fuel [72]. Instead, liquefaction is potentially capable of producing a liquid with considerably lower oxygen content [55,73].

One of the most promising thermochemical liquefaction processes is hydrothermal liquefaction (HTL). HTL is a technology that is capable of efficiently processing wet and dry biomass without restriction of lipid content, from lignocellulosics to organic waste. The product generated in this process is called bio-crude, which is the renewable equivalent to oil, since it is an energy-dense intermediate that can be upgraded to a variety of liquid fuels [7,74]. HTL generates bio-crude from organic matter thanks to specific characteristics, such as the presence of water in hydrothermal conditions, with temperatures ranging from 250 to 450 °C and pressures between 100 and 300 bar. Under these conditions, the water remains in a liquid state or in a very dense supercritical state [12,55,71,75,76,77,78,79]. This technique uses the specific characteristics of compressed hot water [76]. Throughout the liquefaction process, the biomass undergoes a series of depolymerization reactions such as hydrolysis, dehydration or decarboxylation, giving rise to insoluble products such as bio-crude oil or bio-carbon. In addition, other products are also generated, such as gases (CO_2_, CO, H_2_ or CH_4_) or soluble organic substances (mainly acids or phenols) [12,71].

Most of the studies that have been carried out to date focus on the reaction mechanism [80,81], including the effect of biomass types, reaction temperature and heating rate [82], the pressure, type and nature of the catalysts or pH modifiers [83], biofuel performance and final product characteristics [73]. In both the reviews carried out by Gollakota et al. [55] and Castello et al. [12], it is possible to find information about the operation of hydrothermal liquefaction based on the conditions mentioned above, as well as the historical evolution of the technology. According to these studies, different types of biomass give rise to different reaction patterns and respond differently to each of the reaction conditions.

Water is the most widely used solvent to carry out organic reactions, since it is considered a safe, economical and environmentally friendly medium. The vapor pressure curve that separates the liquid and gas phases ends at the so-called critical point (374 °C, 221 bar) [56]. From this point on, the properties of the water can be modified without any phase changes. This results in the supercritical state, which refers to the temperature and pressure zone at the critical point at which water acts as a reactant and a catalyst. In this state, properties of water, such as ionic product, density, viscosity, and dielectric constant, vary rapidly, becoming a liquid without phase limits of high miscibility. Thanks to these properties, supercritical water becomes an apolar solvent for most organic reactions [55,84]. Despite this, in most studies in which hydrothermal liquefaction is analyzed, subcritical conditions (350 °C and >200 bar) have been preferred, i.e., conditions that limit the supercritical state in which the properties of water have changed enough to facilitate liquefaction: the dielectric constant has been reduced by approximately 80% allowing better solubility of apolar compounds.

The elemental composition of the biofuel generated by hydrothermal liquefaction varies as a function of the reaction temperature. The hydrogen content remains constant, but the percentage of oxygen is lower as the process temperature increases, since the increase in temperature favors deoxygenation. The percentage of carbon is higher at higher temperatures. Therefore, the H/C and O/C ratios of the biocrude decrease with the increase in temperature, which is consistent with the increase in the calorific value of the fuel observed (see Table 4). However, production decreases at high temperatures, resulting in less biofuel. Therefore, depending on the needs (quantity or quality), one temperature or another may be of interest, since the ideal temperature to obtain maximum quality fuel is not the ideal temperature to obtain maximum production. It has also been suggested that the heating rate could be an important parameter in hydrothermal liquefaction. In addition, evidence has been found that indicates that the shorter the time in which the reaction is at maximum temperature, the greater the bio-crude production [71,76,77,83].

In subcritical conditions, temperature is the dominant parameter, it becomes less decisive in conditions close to or above the supercritical point [12]. One of the main advances that have been made in the field of hydrothermal liquefaction in recent years is to characterize the effect of pressure in these types of conditions [87,88]. High pressures modify the properties of water, favoring liquefaction over gasification. For example, at a temperature of 400 °C and a pressure of 350 bar, the ionic product is practically the same as at 350 °C and 250 bar. This is interesting, since the high temperatures favor the reactions and their kinetics, achieving greater deoxygenation [12].

The type of biomass used is an important parameter to consider when carrying out hydrothermal liquefaction, since biomass can be made up of different components, which react differently to hydrotreatment. Among the types of biomass that are most commonly used in hydrothermal liquefaction, dry lignocellulosic biomass (composed of cellulose, hemicellulose and lignin) and wet biomass from algae (proteins) and oils (lipids) can be highlighted [55]. According to literature, the components that can most efficiently be converted into biofuel by hydrothermal liquefaction are lipids, proteins and carbohydrates. Among these three components, lipids are the ones that give the best results and carbohydrates the ones that give the poorest results [71].

OFUSW, WWTP, and biomethanization digestate have been considered as low-value materials in the past. Recent studies consider that they have been wasted since their value as an energy source has been increasing, and today, they are considered very valuable resources for the generation of biofuels [41,89,90]. In terms of chemical composition, biomass from bio-waste is highly varied [41]. The chemical compositions of the most commonly used bio-wastes are summarized in Table 5. The waste from WWTP contains a great variety of organic waste, such as oils and fats, that can be used to generate biofuels [49]. In addition, microorganisms, such as algae or bacteria, that grow in this type of wastewater can also be used [89]. Waste from WWTP has also been shown to be a good candidate for bio-crude production due to their cheap price, availability and reasonable calorific value [91]. OFUSW is a biomass with a high lignocellulosic content that is also present in other sources of energy such as food waste [44].

**Figure 2:**
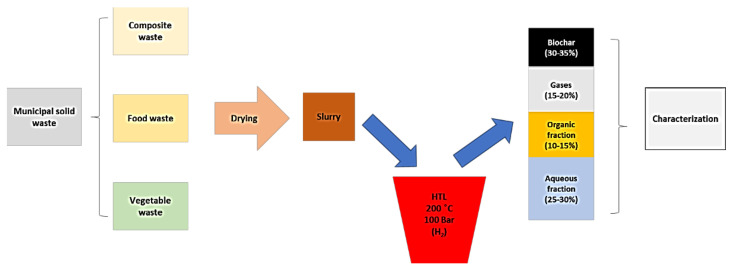
Descriptive diagram of the hydrothermal liquefaction (HTL) process from urban solid waste. Adapted from reference [90].

A descriptive scheme of the experimental methodology for the treatment of urban solid waste by hydrothermal liquefaction is included in Figure 2. The initial fraction of urban solid waste comes from three main sources of waste, namely food waste, agroforestry waste, and compound waste. It is a wet raw material for which a drying pre-treatment is carried out first and the product obtained constitutes the hydrothermal liquefaction feed. Specifically [92], HTL experiments were carried out near the subcritical regions of water (200 °C and 100 bar) in order to obtain a reaction time of 60 min from hydrogen-induced reducing conditions. Several variants of the process were studied and basically four main products were obtained: HTL aqueous fraction (45–50%, HTLAF), organic fraction (45–50%, HTLOF), gas fraction (15–20%, HTLG), and solid fraction of biochar (30–35%, HTLBC) [92,93].

Another parameter that influences the liquefaction process is the catalyst. The use of catalysts in hydrothermal liquefaction can lower the pressure and temperature of the reaction while improving the yield and reducing the solid waste produced. Homogeneous acid or base catalysis is widely used in HTL. Regarding acid catalysis, the most used catalysts for biomass HTL are formic acid, acetic and other weak acids as catalyst solvents, which leads to a bio-oil with a high oxygen content [93]. Although strong acids appear to be effective catalysts, their industrial application is hampered by their strong corrosivity. The basic catalysts are considered better catalysts, specifically, those that are in the form of alkaline salts, such as Na_2_CO_3_, K_2_CO_3_, NaOH, or KOH. A disadvantage of alkaline catalysts is that they can inhibit the dehydration of biological molecules and promote the decarboxylation process due to an increase in the pH of the liquid phase [71]. Many heterogeneous catalysts, mainly in the form of various metals, such as Pd, Ru, Pt, Mo, Ni, Co, and Ni, on SiO_2_, Al_2_O_3_, and zeolite, have been investigated. However, some drawbacks have been found that limit their application, such as poisoning, sintering, or intraparticular diffusion [71]. Biller et al. [94] carried out an investigation of the HTL of humid biomass with heterogeneous catalysts Co/Mo, Ni/Al and Pt/Al. In their conclusions, it appears that these catalysts promote the calorific value, the oxygen content, and the performance of the bio-oil. In addition, it has been reported that they also play a very important role in reducing the nitrogen content of the bio-oil. Wang et al. [95] studied the effect of HTL of microalgae *Nannochloropsis* (NAS) over various transition metal/TiO_2_ catalysts. As can be seen in Table 6, with the Ni/TiO_2_ catalyst, a liquefaction conversion of 85.19% and a bio-crude yield of 42.40% were obtained, which was 10% higher than the blank experiment.

#### Operation Mode: Batch Reactors and Continuous Hydrothermal Liquefaction

Hydrothermal liquefaction has been widely studied in batch type discontinuous reactors [73]. By means of this procedure, any type of material can be screened, and a wide range of process conditions can be evaluated [83,96]. Another great advantage of the process is that relatively high concentrations of dry matter can be obtained. In addition, there are no complications, e.g., plugging in the pipes or difficulties in pressurizing and pumping the raw material. However, a number of important drawbacks have been described concerning continuous hydrothermal liquefaction [12]:

Thermal fugacity: In batch type reactors, the process conditions are not constant, because the system has to go from ambient conditions to the desired temperature. This transience makes it difficult to separate the effects of temperature and time. The faster the heating, the less this problem is present.

Difficulty decoupling temperature and pressure: In most batch experiments, pressure is obtained by heating the reagents. Pre-pressurizing the system with an inert gas can partially solve this problem. However, the elevated pressures increase the solubility of the inert gas, reducing the usefulness of the pre-pressurization. In a continuous system, pressure and temperature can be controlled completely independently.

Initial contact pattern: In a batch reactor, the reactants are usually mixed by stirring the reactor itself or thanks to an impeller. This contact pattern is different from that observed in continuous reactors. That is, in continuous flow reactors, new reagents are continuously supplied while products are removed. In a continuous tubular reactor, the flow pattern (laminar or turbulent) can significantly change the results of the process.

Difficulty for its implementation at the industrial level: The industrial use of batch reactors is normally justified only for the production of high added value products, often produced in limited quantities. This is definitely not the case for biofuel production, as the goal is to produce large quantities. Furthermore, hydrothermal liquefaction requires extensive optimization to reduce the energy consumption of the process, which can only be carried out effectively in a continuous configuration.

#### Biofuel Optimization

In general, it can be said that the most severe conditions cause a high degree of deoxygenation and denitrogenation. At the same time, a higher quality of the bio-oil is associated with a significant decrease in its yields. The best results, considering both quantity and quality, are obtained when using a system as a catalyst based on Co/Mo [97].

**Figure 3:**
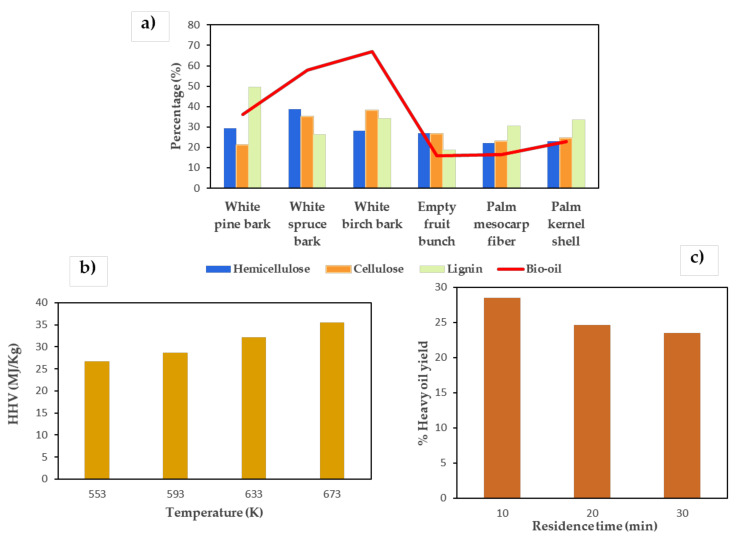
Effect of type of biomass (**a**) [99,100], temperature (**b**) [85,86] and residence time (**c**) [101] on HTL products.

In a study to obtain bio-crude from *Nannochloropsis algae* in a continuous process, López Barreiro et al. [7] report different yields depending on the catalyst used. The best results in terms of deoxygenation were obtained with a Pt/Al_2_O_3_ catalyst reaching 1.6% oxygen and 63.5% yield. The highest yield values (96.6%) were obtained with a NiMo/Al_2_O_3_ catalyst. There are some less important parameters that have a direct relationship with the yield and quality of the bio-crude. They are as follows:

Type of biomass: Biomass with a higher percentage of cellulose produces a higher conversion than that with a higher proportion of lignin (see Figure 3a) [98].

Effect of temperature: Temperature is one of the decisive parameters of the HTL process. When working at subcritical temperatures, the break of chemical bonds and the depolymerization of the biomass are increased. As a consequence, the concentration of free radicals and the probability of repolymerization of molecular fragments are also increased to increase the total yield of the bio-crude and the HHV (see Figure 3b). When the temperature approaches or exceeds the critical point of water, the repolymerization of intermediate products increases, therefore the performance decreases [71].

Heating rate: Slow heating rates generally lead to the formation of carbon residues due to the repolymerization of intermediates, while fast heating rates lead to the formation of gaseous products due to redecomposition reactions. Therefore, proper heating rates are an important parameter in the HTL process. More research on heating rates is necessary to fully understand the mechanism of this conversion process [71].

Time of retention/residence: A large number of investigations have been carried out on the effect of residence times on the yield of bio-crudes. It is expected that the short retention times produce a large amount of bio-oil, and conversely, a long retention time causes the repolymerization of the intermediate products, which therefore reduces the yield of the biocrude (see Figure 3c) [71].

Particle size: A reduction in the size of the particles seems to indicate an improvement in the performance of the bio-oil, as well as a decrease in the amount of solid waste. An optimal particle size for the HTL process is considered to be between 4 and 10 mm [71].

Although hydrothermal liquefaction is a hopeful process with great potential, the bio-crude generated usually has a high content of heteroatoms, mainly oxygen, nitrogen and sulfur. Therefore, in its original form, bio-crude is not compatible with most of the uses for which it is intended in the transport sector and requires improvement or optimization [102]. Oxygen reduces the calorific value and energy density of bio-crude, while nitrogen and sulfur corrode equipment and pollute the atmosphere [103].

One of the most promising techniques for improving the quality of biocrude and its conversion to hydrocarbons is hydrotreating. This process, which represents a serious technological challenge, removes heteroatoms from the bio-crude in the presence of heterogeneous catalysts like CoMo/γ-Al_2_O_3_ or NiMo/γ-Al_2_O_3_ at high temperatures and pressures (300–450 °C and 30–170 bar) [74]. The presence of considerable amounts of oxygenated compounds makes the bio-crude obtained by liquefaction unstable under severe conditions, which means that it could be susceptible to polymerization or condensation reactions, as observed in bio-oils obtained by pyrolysis [104]. Hydrotreating removes oxygen through dehydration, decarboxylation, and decarbonylation reactions and removes nitrogen mainly in the form of ammonia. Nitrogen-containing compounds found in biocrude increase chemical complexity and lead to higher H_2_ consumption, as they are recalcitrant to denitrogenation. Nitrogen compounds can be difficult to remove, so more severe conditions are required in terms of temperature and partial pressure of H_2_ to be able to remove them [105,106,107].

**Figure 4:**
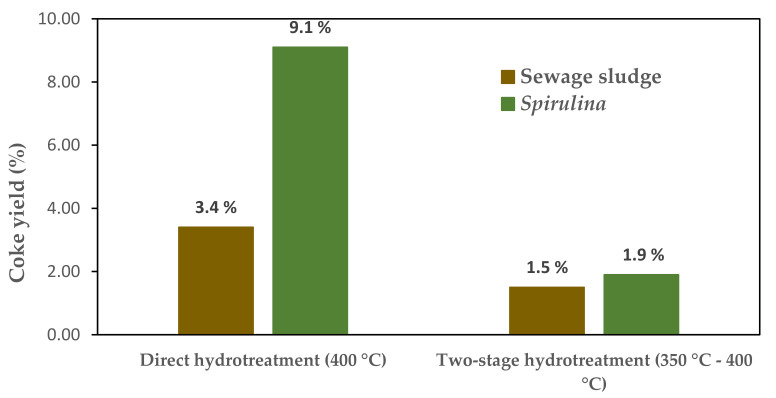
Comparison of coke yeld obtained after direct hydrotreating at 400 °C and two-stage hydrotreating. Reproduced from reference [74].

As bio-crudes obtained by liquefaction are generally more stable than bio-oils obtained by pyrolysis due to their lower oxygen content, less attention has been paid to the problem of the formation of coke at such high temperatures (400 °C) [74]. That is to say, severe thermal conditions are associated with effective hydrodenitrogenation. However, these conditions promote unwanted polymerization or coking reactions, due to the presence of a considerable amount of reactive oxygen-containing functional groups. Haider et al. [74] have proposed the use of a multi-stage hydrotreatment to overcome this problem. According to their study, by performing a first hydrotreating stage at 350 °C, practically complete deoxygenation is achieved, resulting in a much lower amount of coke (see Figure 4). As a next step, they carried out a second stage at a higher temperature at which a denitrogenation of 92% was obtained.

## 5. Summary and Future Perspectives

Various studies have analyzed the production of biofuels from the organic fraction of urban solid waste and sludge from treatment plants. The results of these investigations seem to indicate that this type of second generation biomass can be a viable energy source, making it possible to reduce waste while generating a biofuel with a high calorific value. According to these works, hydrothermal liquefaction combined with the appropriate previous and subsequent treatments, is the best method to value this type of bio-waste. Along these lines, there are investigations that have been carried out at the industrial level that ensure that continuous hydrothermal liquefaction is an effective technology for the processing of sludge from sewage treatment plants. Although it has been observed that depolymerization is greater in the subcritical region of water (200 °C and 100 bar), the effects of these two parameters, as well as the influence of other factors, such as the presence of hydrogen and the residence time for HTL of a given raw material, need to be further studied. In addition, it is necessary to establish pilot plants as well as to investigate and optimize the reaction pathways of HTL, using both model compounds and real biomass. Furthermore, theoretical models based on computational fluid dynamics can be an important tool, since they allow for the analysis of complex processing parameters, such as the thermodynamic properties of the bio-crude or the effect of viscosity [55]. Finally, it must be taken into account that, in addition to subcritical water, large amounts of organic solvents are still used to recover bio-crude in subsequent processing, which reduces the sustainability of the process. Thus, it is crucial to develop a solvent-free HTL technique.

## Figures and Tables

**Table 1:** Comparison of the main characteristics of diesel, biodiesel and green diesel [26,27,29].

	Conventional Diesel	Biodiesel	Green Diesel
Density (kg/m^3^)	840	880	780
Carbon (wt%)	86.8	76.2	84.9
Oxygen (wt%)	0	10–12	0
Sulfur (mg/kg)	<10	<1	<1
Heating value (MJ/kg)	43	38	44
Cetane index (%)	40–50	50–65	70–90
CO_2_ emissions (kg/MJ)	0.08	0.06	0.40

**Table 2:** Average composition of USW [47].

Material	Composition (%)	Material	Composition (%)
Food waste	41.52	Textile fibers	0.77
Garden waste	12.65	Wood	0.64
Paper and paperboard	10.36	Clay	0.52
Plastic components	8.47	Cans	0.45
Disposable diapers	6.62	Polymers (polyurethane, polypropylene and polystyrene)	0.45
Crystals	4.05	Cork	0.4
Construction materials rubble	1.2	Electric batteries	0.05
Iron	0.93	Others	10.92

**Table 3:** Advantages and disadvantages of combustion, gasification and pyrolysis [63].

Technology	Advantages	Disadvantages
Combustion	Volume reduction Controlled emissions Use of heat for power generation Silent and odorless	High initial investment High costs to avoid pollution by emissions Possible generation of harmful products for health (dioxins, furans, heavy metals) Possible conflicts with programs aimed at reducing waste generation It requires more energy if waste with high percentages of humidity is treated
Gasification	Formation of a synthesis gas with various uses (production of electricity, fuel, production of chemicals) Prevents the formation of dangerous nitrogenous, halogenated and sulfur compounds	Limited large-scale experiences Use of resources that are preferably destined for recycling It requires more energy if waste with high percentages of humidity is treated.
Pyrolysis	Use of by-products in other processes It allows to generate specific products according to the operating conditions Prevents formation of dangerous nitrogenous, halogenated and sulfur compounds	High initial investment cost Unwanted products can be obtained Does not have large-scale facilities The most desired resources are usually separated for recycling purposes It requires more energy if waste with high percentages of humidity is treated

**Table 4:** The effect of temperature on the elemental composition, atomic ratios and Higher Heating Value (HHV) of biofuel [85,86].

Temperature (°C)	H (%)	O (%)	C (%)	H/C	O/C	HHV (MJ/kg)
280	7.36	24.30	67.03	1.32	0.27	26.75
320	7.65	22.13	68.77	1.33	0.24	28.63
360	7.73	18.14	72.81	1.27	0.19	32.16
400	7.36	14.07	77.22	1.14	0.14	35.48

**Table 5:** Chemical compositions of various biomass residues [45].

Biomass Waste	Type of Biomass	Cellulose (%)	Hemicellulose (%)	Lignin (%)
Agriculture residues	Corn stover	38–40	28	7–21
	Barley straw	33	26	19
Forestry residues	Poplar saw dust	44	19	25
	Eucalyptus	54	18	21
	Municipal greening waste	33	26	19
Energy crops	Miscanthus x giganteus	41–53	20–25	20–23
	Napier grass (Pennisetum purpureum)	40–50	20–40	10–25
	Pennisetum pedicellatum	32	23	3

**Table 6:** Effect of transition metal/TiO_2_ catalyst on liquefaction conversion and bio-crude yield [95].

	Co	Fe	Mn	Mo	Ni	Blank
Liquefaction conversion (%)	75.97	78.72	82.56	84.33	85.19	79.31
Biocrude yield (%)	32.73	29.10	31.63	35.86	42.40	30.10

## Data Availability

Not applicable.
